# Osteoporosis, Fear of Falling, and Restrictions in Daily Living. Evidence From a Nationally Representative Sample of Community-Dwelling Older Adults

**DOI:** 10.3389/fendo.2019.00646

**Published:** 2019-09-26

**Authors:** Felix Meyer, Hans-Helmut König, André Hajek

**Affiliations:** Department of Health Economics and Health Services Research, Hamburg Center for Health Economics, University Medical Center Hamburg-Eppendorf, Hamburg, Germany

**Keywords:** aged, cross-sectional studies, fear of falling, Germany, osteoporosis, restrictions

## Abstract

**Background:** There is a lack of studies examining the relationship between osteoporosis and fear of falling as well as the association of osteoporosis and restrictions in daily life due to fear of falling. Thus, the aim of this study was to investigate whether there is an association between the presence of osteoporosis and fear of falling as well as restrictions in daily life due to fear of falling.

**Methods:** Cross-sectional data were used from a population-based sample of community-dwelling individuals in the second half of life (40 to 95 years; *n* = 7,808) in Germany. GP-diagnosed osteoporosis was used. Fear of falling as well as the restrictions in daily life due to fear of falling were collected in self-administered questionnaires. Multiple regression models controlling for sociodemographic, lifestyle, and health-related variables were used to determine the association between osteoporosis and the outcome measures.

**Results:** Logistic regressions showed that osteoporosis was associated with increased fear of falling in the total sample and in both sexes. In addition, regressions showed that osteoporosis was associated with restrictions in daily life due to fear of falling in the total sample and in women, but not in men.

**Conclusions:** The present study showed that osteoporosis is associated with fear of falling and with restrictions in daily life due to fear of falling. Because effective interventions to treat the fear of falling are available, our study might help to address this target group more accurately.

## Introduction

Osteoporosis, defined as a skeletal disorder characterized by compromised bone strength, is a frequent problem among older adults (1). Bone strength is primarily associated with bone density and bone quality. Regarding this, bone density is defined as grams of mineral per volume. Main associations of bone quality are architecture, turnover, damage accumulation, and mineralization (1). The prevalence of osteoporosis differed by age and sex. In general, the prevalence increased with age and was higher in women (2). It is estimated that over 200 million people worldwide suffer from osteoporosis. Further prognosis shows that until 2025 over 500 million people might suffer from this disease (3).

In the time period from 2009 to 2050 the proportion of people aged 50 years and older is expected to increase from 39 to more than 50% of the total German population (4), underlining the importance of identifying the consequences of osteoporosis.

The importance of the topic is also underlined by the fact, that osteoporosis is associated with falls, and fractures. Individuals with osteoporosis have a higher risk of falls, due to muscle weakness, spine kyphosis, or decreased postural control (5, 6). Low bone density due to osteoporosis is a main reason that falls easily result in fractures (7). Moreover, Delbaere et al. (8) show that fear of falling is strongly associated with frequent falls.

Thus far, most studies are restricted to the relation of osteoporosis and fear of falling. For example, the study by Resnick et al. found a positive correlation of osteoporosis and fear of falling (9). However, there is a lack of studies examining the relationship between osteoporosis and fear of falling as well as the association of osteoporosis and restrictions in daily life due to fear of falling.

For these reasons, the aim of this study was to investigate the gap between osteoporosis and restrictions in daily life due to fear of falling. In accordance with previous studies, we hypothesize that (1) fear of falling is positively associated with osteoporosis. Equally, we hypothesize that (2) restrictions in daily life due to fear of falling are positively associated with osteoporosis.

This knowledge is helpful for addressing the target group more accurately by characterizing people with fear of falling in detail.

## Methods

### Sample

We used cross-sectional data from the fifth wave (2014) of the German Aging Survey (DEAS; “Deutscher Alterssurvey”). The DEAS is a large, population-based sample of individuals aged ≥40 years. The DEAS started with the first wave in 1996. Follow-up waves comprising cross-sectional and panel samples took place in 2002 (second wave), 2008 (third wave), 2011 (fourth wave), as well as in 2014 (fifth wave). The DEAS used a cohort-sequential design, consisting of cross-sectional samples, and longitudinal samples. The cross-sectional samples included first time participants, whereas the panel samples included individuals already interviewed before. The main part of the DEAS is divided into a personal interview and an additional self-administered questionnaire. Klaus et al. provided further details regarding the DEAS study (10).

We focused on the fifth wave of the DEAS study because our target dimensions were only assessed in this wave. The cross-sectional sample of the fifth wave consisted of the panel participants (*n* = 4,352), who had previously participated in the study (response rate 61%), and a newly drawn cross-sectional sample (*n* = 6,003), who were participating in the DEAS study for the first time (response rate 25%). In total, 10,355 participants were interviewed. Written consent was obtained from all participants. It was not necessary to obtain permission from ethics committees, as the criteria for requiring an ethics statement were not met.

### Dependent Variables

Fear of falling was self-assessed in the additional self-administered questionnaire. Therefore, the participants answered the question: “The following questions are about involuntary falls: Were you afraid that you might fall during the past 12 months?” The offered possibilities to answer were “yes” or “no.” This is a common way of quantifying fear of falling (11).

Furthermore, for measuring restrictions in daily life due to fear of falling the participants were asked the following question: “Do you ever limit your activities, for example, what you do, or where you go, because you are afraid of falling?” The interviewees were offered the possibility to answer with “yes” or “no.”

### Independent Variables

Osteoporosis was assessed according to a list of various illnesses diagnosed by a physician. The participants' possibilities to answer were “yes” or “no.” To put it in other words: Individuals were asked to identify from a list of several illnesses (heart attack; high blood pressure, or hypertension; high blood cholesterol; stroke or cerebral vascular disease; diabetes or high blood sugar; chronic lung disease; arthritis, including osteoarthritis, or rheumatism; cancer or malignant tumor; stomach or duodenal ulcer, peptic ulcer; Parkinson's disease; cataracts; hip fracture or femoral fracture) which illnesses they had been formally diagnosed with by their doctor. The list is based among others on the Charlson Comorbidity Index. The selection of diseases was also based on several sources (12).

### Control Variables

Age and sex were included in the regression model. In addition, employment status was operationalized as employed, retired and other: not employed. Marital status was collected as married and living with a spouse, married and living separately, divorced, widowed, and single. We also included individual monthly net equivalent income (OECD scale). Moreover, it was adjusted for the lifestyle factors of alcohol consumption, and physical activity. We distinguished between drinking alcohol using “daily,” “several times a week,” “once a week,” “one to 3 times a month,” “less frequently,” and “never.” The same categories were used to measure physical activity. In addition, the number of physical illnesses such as cardiovascular disease or cancer, as well as self-rated health were included in the regression model. The range of the number of physical illnesses is between 0 and 11 and self-rated health ranges from 1 = “very good” to 5 = “very bad.”

### Statistical Analysis

First, sample characteristics were reported stratified by osteoporosis. In addition, multiple logistic regressions were used to model the relation between osteoporosis and fear of falling as well as restrictions in daily life due to fear of falling, controlling for several potential confounders. The model assumptions for the logistic regressions were checked. Moreover, we tested for multicollinearity (using the variance inflation criterion). Across the regressions, it was found that the largest variance was 1.47, indicating that we do not have a problem with multicollinearity.

The level of statistical significance was set to *p* < 0.05. All statistical analyses were conducted using Stata 15.0 (Stata Corp, College Station, TX, USA).

## Results

### Bivariate Analysis

In sum, 7,213 participants reported whether osteoporosis was diagnosed or not (average age equaled 64.4 ± 11.2, 40 to 95 years). About 3,641 of these respondents were female (50.5%), 5,029 individuals were married, living together with spouse (69.7%). In total, 512 respondents (116 men; 396 women) reported that osteoporosis was diagnosed (7.1%; in men: 3.3%; in women: 10.9%). Respondents with diagnosed osteoporosis reported higher fear of falling as well as higher restrictions in daily life through fear of falling. Please see Table 1 for further details.

**Table 1:** Characteristics, stratified by diagnosed osteoporosis (No; Yes) (*n* = 7,213).

	**Absence of osteoporosis (*n* = 6,701)**	**Presence of osteoporosis (*n* = 512)**	***p*-value**
Age in years: Mean (SD)	64.0 (11.2)	70.0 (9.9)	<0.001
Employment status: *N* (%)			<0.001
-Employed	2,575 (97.2)	75 (2.8)	
-Retired	3,546 (90.2)	386 (9.8)	
-Other not employed	580 (91.9)	51 (8.1)	
Marital status; *N* (%)			<0.001
-Married, living together with spouse	4,715 (93.8)	314 (6.2)	
-Other	1,986 (90.9)	198 (9.1)	
Monthly net equivalent income; Euro (SD)	1,967.4 (1,410.9)	1,673.4 (964.4)	
Consumption of alcohol; *N* (%)			<0.001
-Daily	826 (94.4)	49 (5.6)	
-Several times a week	1,687 (95.3)	83 (4.7)	
-Once a week	1,080 (94.7)	61 (5.3)	
-One to three times a month	810 (91.3)	77 (8.7)	
-Less frequently	1,579 (91.6)	144 (8.4)	
-Never	719 (0.88)	98 (0.12)	
Physical activity; *N* (%)			<0.001
-Daily	536 (89.9)	60 (10.1)	
-Several times a week	1,838 (93.9)	120 (6.1)	
-Once a week	1,215 (92.1)	104 (7.9)	
-One to three times a month	510 (94.3)	31 (5.7)	
-Less frequently	810 (95.5)	38 (4.5)	
-Never	1,792 (91.9)	159 (8.1)	
Total number of physical illnesses; Mean (SD)	2.5 (1.8)	3.5 (2.0)	<0.001
Self-rated health; Mean (SD)	2.5 (0.8)	2.9 (0.9)	<0.001
Fear of falling; *N* (%)			
-Yes	1,061 (82.6)	223 (17.4)	<0.001
-No	5,640 (95.1)	289 (4.9)	<0.001
Restrictions due to fear of falling; *N* (%)			
-Yes	458 (80.5)	111 (19.5)	<0.001
-No	6,234 (94.0)	398 (6.0)	<0.001

### Regression Analysis

After controlling for various covariates, multiple logistic regressions (Table 2) showed that increased fear of falling was associated with osteoporosis [OR: 2.40 (95%–CI: 1.95–2.96)] in the total sample and in both sexes [men, OR: 1.64 (1.03–2.62), women, OR: 2.04 (1.60–2.60)]. With regard to covariates, only self-rated health, and number of physical illnesses were consistently associated with fear of falling in the total sample, and in both sexes.

**Table 2:** Determinants of fear of falling and restrictions in daily life due to fear of falling.

	**Fear of falling**			**Restriction in daily life (due to fear of falling)**		
	**(1)**	**(2)**	**(3)**	**(4)**	**(5)**	**(6)**
**Independent variables**	**Total sample**	**Men**	**Women**	**Total sample**	**Men**	**Women**
Presence of osteoporosis (ref.: absence)	2.40^***^ (1.95–2.96)	1.64^*^ (1.03–2.62)	2.04^***^ (1.60–2.60)	1.91^***^ (1.47–2.49)	1.56 (0.88–2.77)	1.82^***^ (1.33–2.48)
Age in years, 2014	1.04^***^ (1.03–1.05)	1.04^***^ (1.03–1.06)	1.04^***^ (1.03–1.06)	1.05^***^ (1.03–1.06)	1.05^***^ (1.03–1.07)	1.05^***^ (1.03–1.07)
Employment Status (ref.: employed)						
-Retired	1.08 (0.85–1.38)	1.12 (0.74–1.68)	1.15 (0.84–1.56)	1.38 (0.95–2.00)	1.41 (0.78–2.56)	1.42 (0.88–2.31)
-Other not employed	1.14 (0.86–1.50)	0.55 (0.29–1.03)	1.26 (0.91–1.75)	1.31 (0.84–2.03)	0.94 (0.41–2.14)	1.41 (0.83–2.42)
Marital status: other (married, living separated from spouse; divorced, widowed, single) (reference: married, living together with spouse)	1.44^***^ (1.25–1.66)	1.24 (0.97–1.59)	1.34^**^ (1.11–1.60)	1.39^**^ (1.14–1.69)	1.36 (0.99–1.88)	1.30^*^ (1.00–1.69)
Monthly net equivalent income	1.00 (1.00–1.00)	1.00 (1.00–1.00)	1.00 (1.00–1.00)	1.00 (1.00–1.00)	1.00 (1.00–1.00)	1.00 (1.00–1.00)
Consumption of Alcohol (ref.: never)						
-Daily	0.75^*^ (0.58–0.97)	0.87 (0.59–1.27)	1.03 (0.68–1.55)	0.88 (0.61–1.26)	0.99 (0.60–1.64)	0.92 (0.49–1.72)
-Several times a week	0.62^***^ (0.49–0.79)	0.58^**^ (0.40–0.84)	0.91 (0.66–1.25)	0.73 (0.52–1.02)	0.67 (0.40–1.11)	0.98 (0.61–1.58)
-Once a week	0.68^**^ (0.52–0.88)	0.61^*^ (0.40–0.93)	0.82 (0.58–1.14)	0.87 (0.60–1.24)	1.00 (0.58–1.73)	0.82 (0.50–1.35)
-One to three times a month	0.92 (0.71–1.20)	0.64 (0.40–1.02)	1.17 (0.84–1.62)	1.31 (0.92–1.86)	1.08 (0.59–1.97)	1.52 (0.97–2.38)
-Less frequently	0.96 (0.78–1.19)	0.83 (0.57–1.21)	0.98 (0.76–1.28)	1.10 (0.83–1.46)	1.10 (0.68–1.79)	1.09 (0.77–1.56)
Physical activity (ref.: never)						
-Daily	1.17 (0.91–1.50)	0.90 (0.59–1.37)	1.23 (0.89–1.70)	0.99 (0.71–1.39)	1.00 (0.59–1.72)	0.92 (0.59–1.43)
-Several times a week	0.79^*^ (0.65–0.96)	0.75 (0.55–1.03)	0.73^*^ (0.56–0.94)	0.69^*^ (0.52–0.92)	0.61^*^ (0.39–0.96)	0.70 (0.48–1.01)
-Once a week	0.97 (0.79–1.18)	0.72 (0.50–1.02)	0.95 (0.74–1.24)	0.72^*^ (0.53–0.96)	0.60^*^ (0.36–0.99)	0.73 (0.50–1.06)
-One to three times a month	0.95 (0.71–1.26)	0.86 (0.55–1.33)	0.99 (0.67–1.47)	0.71 (0.46–1.11)	0.96 (0.54–1.72)	0.51 (0.26–1.00)
-Less frequently	0.88 (0.69–1.11)	0.70 (0.48–1.00)	1.09 (0.78–1.51)	0.88 (0.63–1.23)	0.84 (0.53–1.35)	0.96 (0.60–1.56)
Number of physical illnesses, 2014	1.24^***^ (1.19–1.29)	1.23^***^ (1.16–1.31)	1.28^***^ (1.22–1.35)	1.22^***^ (1.16–1.28)	1.25^***^ (1.16–1.35)	1.21^***^ (1.13–1.30)
Self-rated health (from 1 = “very good” to 5 = “very bad”)	1.92^***^ (1.76–2.11)	2.09^***^ (1.81–2.41)	1.95^***^ (1.73–2.20)	2.53^***^ (2.23–2.86)	2.46^***^ (2.04–2.96)	2.65^***^ (2.24–3.14)
Constant	0.00^***^ (0.00–0.00)	0.00^***^ (0.00–0.00)	0.00^***^ (0.00–0.00)	0.00^***^ (0.00–0.00)	0.00^***^ (0.00–0.00)	0.00^***^ (0.00–0.00)
Observations	7,213	3,572	3,641	7,213	3,571	3,642
Pseudo R^2^	0.186	0.185	0.202	0.231	0.222	0.244

Results of multiple logistic regressions (German Aging Survey, fifth wave). Odds ratios were reported; 95 % Confidence intervals in parentheses;

*** *p < 0.001*,

** *p < 0.01*,

* *p < 0.05*.

Moreover, increased restrictions in daily life due to fear of falling were associated with osteoporosis [OR: 1.91 (1.47–2.49)] in the total sample, in women, but not in men [men, OR: 1.56 (0.88–2.77), women, OR: 1.82 (1.33–2.48)]. Regarding the covariates, only self-rated health and number of physical illnesses were consistently associated with increased restrictions in daily life due to fear of falling in the total sample, and in both sexes.

Furthermore, regressions were stratified by age (40 to 64 years; 65 years and over). Among individuals 40 to 64 years, regressions showed that osteoporosis was associated with increased fear of falling in the total sample [OR: 2.95 (1.98–4.39)], and in women [OR: 2.86 (1.81–4.51)], but not in men [OR: 1.77 (0.72–4.36)]. Increased restrictions in daily living due to fear of falling were not associated with osteoporosis [total sample, OR: 1.39 (0.76–2.56), men, OR: 0.72 (0.18–2.90), women, OR: 1.61 (0.80–3.23)].

Among individuals 65 years and over, regressions showed that osteoporosis was associated with increased fear of falling in the total sample [OR: 2.19 (1.71–2.81)] and in women [OR: 1.74 (1.30–2.33)], but not in men [OR: 1.62 (0.93–2.80)]. Increased restrictions in daily living due to fear of falling were associated with osteoporosis in the total sample [OR: 2.08 (1.54–2.81)], and in both sexes [men, OR: 1.97 (1.03–3.76), women, OR: 1.89 (1.33–2.68)].

## Discussion

### Main Findings

Based on a large nationally representative sample of community-dwelling individuals in the second half of life, the aim of this study was to investigate whether there is an association between the presence between osteoporosis and fear of falling as well as restrictions in daily life due to fear of falling. Multiple logistic regression analysis revealed that osteoporosis was associated with higher fear of falling in the total sample as well as in women and men after adjusting for socio-demographic factors, various lifestyle factors, self-rated health and number of physical illnesses. Moreover, osteoporosis was associated with restrictions in daily life due to fear of falling in the total sample as well as in women but not in men after adjusting for the same set of covariates.

### Relation to Previous Research

To our best knowledge, the current study is the first one, which investigated the relation between osteoporosis, and fear of falling as well as *restrictions in daily life due to fear of falling*. Most of the existing studies only consider the relation between osteoporosis and fear of falling. As an example a paper by Resnick et al. showed that diagnosed osteoporosis is associated with fear of falling (9). This is in accordance with our study and appears plausible because people with osteoporosis have lower bone mass than healthy individuals and therefore are more instable (13). Because of the instability, these people might have an increased fear of falling.

However, by indicating a gap in the previous research, this study further assessed the association between diagnosed osteoporosis and *restrictions in daily life due to fear of falling*. It appears very plausible that diagnosed osteoporosis and *restrictions in daily life due to fear of falling* are positively associated. A possible explanation for our findings might be the fact that individuals with osteoporosis are more fragile and have higher balance deficits (13). Therefore, they might have higher *restrictions in daily life due to fear of falling*.

The non-significant relationship between diagnosed osteoporosis and *restrictions in daily life due to fear of falling* in men might be explained by the fact that in general men have a different health risk behavior than women. Men are more likely than women to adopt behaviors that increase their risks regarding health (14). However, this should be further investigated in future studies. The lack of association may also be explained by the lack of statistical power.

### Strengths and Limitations

This is one of a few studies that investigated not only the association between osteoporosis and fear of falling but also restrictions in daily living because of fear of falling. In contrast to existing studies which are mainly restricted to rather specific samples, our results provide new insights by using a large nationally representative sample of community-dwelling individuals aged 40 and over (10). In contrast to previous studies which are restricted to osteoporosis samples, our study compared individuals with osteoporosis with a healthy comparison group. GP-diagnosed osteoporosis was used as main independent variable.

The limitations of the present study are particularly related to potential sample selection bias. It can be observed among the oldest age groups. However, it has been demonstrated that selectivity effects are small in the German Aging Survey and the distribution of sociodemographic factors (e.g., family status or household size) are close to the distribution within the German population (15). Moreover, this is a cross-sectional study. The causal direction of this relationship (e.g., fear of falling and osteoporosis) could be argued to be reciprocal. Limitations also arise from the fact that self-reported single-item measures were used to assess fear of falling and restrictions in daily life due to fear of falling. The prevalence of osteoporosis is rather low in the DEAS study. Therefore, we cannot dismiss the possibility that this is underestimated. In the DEAS study, data about the pharmacological treatment of osteoporosis is missing.

### Conclusion

Findings of the present study stress the relation between osteoporosis and fear of falling as well as restrictions in daily life due to fear of falling. Because effective interventions to treat the fear of falling are available, our study might help to address this target group more accurately.

Future longitudinal studies are needed to validate the findings of the present cross-sectional study and to better understand the relationship of our variables of interest. Moreover, there are large issues of postmenopausal osteoporosis (16, 17). This should be investigated in further studies.

## Data Availability Statement

The data used in this study are third-party data. The anonymized data sets of the DEAS (1996, 2002, 2008, 2011, and 2014) are available for secondary analysis. The data has been made available to scientists at universities and research institutes exclusively for scientific purposes. The use of data is subject to written data protection agreements. Microdata of the German Aging Survey (DEAS) is available free of charge to scientific researchers for non-profitable purposes. The FDZ-DZA provides access and support to scholars interested in using DEAS for their research. However, for reasons of data protection, signing a data distribution contract is required before data can be obtained. Please see for further Information (data distribution contract): https://www.dza.de/en/fdz/access-to-data/formular-deas-en-english.html.

## Ethics Statement

Please note that an ethical statement for the DEAS study was not necessary because criteria for the need of an ethical statement were not met (risk for the respondents, lack of information about the aims of the study, examination of patients). This is an accordance with the German Research Foundation-guidelines (Deutsche Forschungsgemeinschaft, DFG) available at: http://dfg.de/foerderung/faq/geistes_sozialwissenschaften/ (only available in German language). The German Centre of Gerontology (DZA) decided that an ethical statement was not necessary. It is worth noting that the DEAS study has a permanent advisory board. Prior to each wave of data collection, the permanent advisory board received detailed information about the sampling method, the consent to participate and the instruments used in the DEAS study. The permanent advisory board concluded that the DEAS study did not need approval from an ethics committee. This procedure is in concordance with local guidelines. Please also see the RatSWD (Principles and Review Procedures of Research Ethics in the Social and Economic Sciences): https://www.ratswd.de/dl/RatSWD_Output9_Forschungsethik.pdf, page 28 (only available in German language). Informed consent was obtained from all individual participants included in the study.

## Author Contributions

FM, H-HK, and AH design and concept of analyses, preparation of data, statistical analysis and interpretation of data, and preparing of the manuscript. All authors critically reviewed the manuscript, provided significant editing of the article, and approved the final manuscript.

### Conflict of Interest

The authors declare that the research was conducted in the absence of any commercial or financial relationships that could be construed as a potential conflict of interest.
